# Supplementary material for: An optimized method for high-quality RNA extraction from distinctive intrinsic laryngeal muscles in the rat model
Source: Sci Rep. 2022 Dec 15;12:21665. doi: 10.1038/s41598-022-25643-y (PMC9755529; doi:10.1038/s41598-022-25643-y)
Supplement: Supplementary file 1 — Supplementary Figures. [file 41598_2022_25643_MOESM1_ESM.pdf]

SUPPLEMENTARY FILES

An optimized method for high-quality RNA extraction from distinctive intrinsic laryngeal muscles in the rat model.

AUTHORS

Angela M. Kemfack, BS[1]; Ignacio Hernández-Morato, PhD[1*]; Yalda Moayedi, PhD[1,2]; Michael J. Pitman, MD[1]

1. Department of Otolaryngology-Head & Neck Surgery, Columbia University College of Physicians and Surgeons, New York, NY

2. Department of Neurology, Columbia University College of Physicians and Surgeons, New York, NY

ORIGINAL GELS OF ELECTROPHEROGRAM

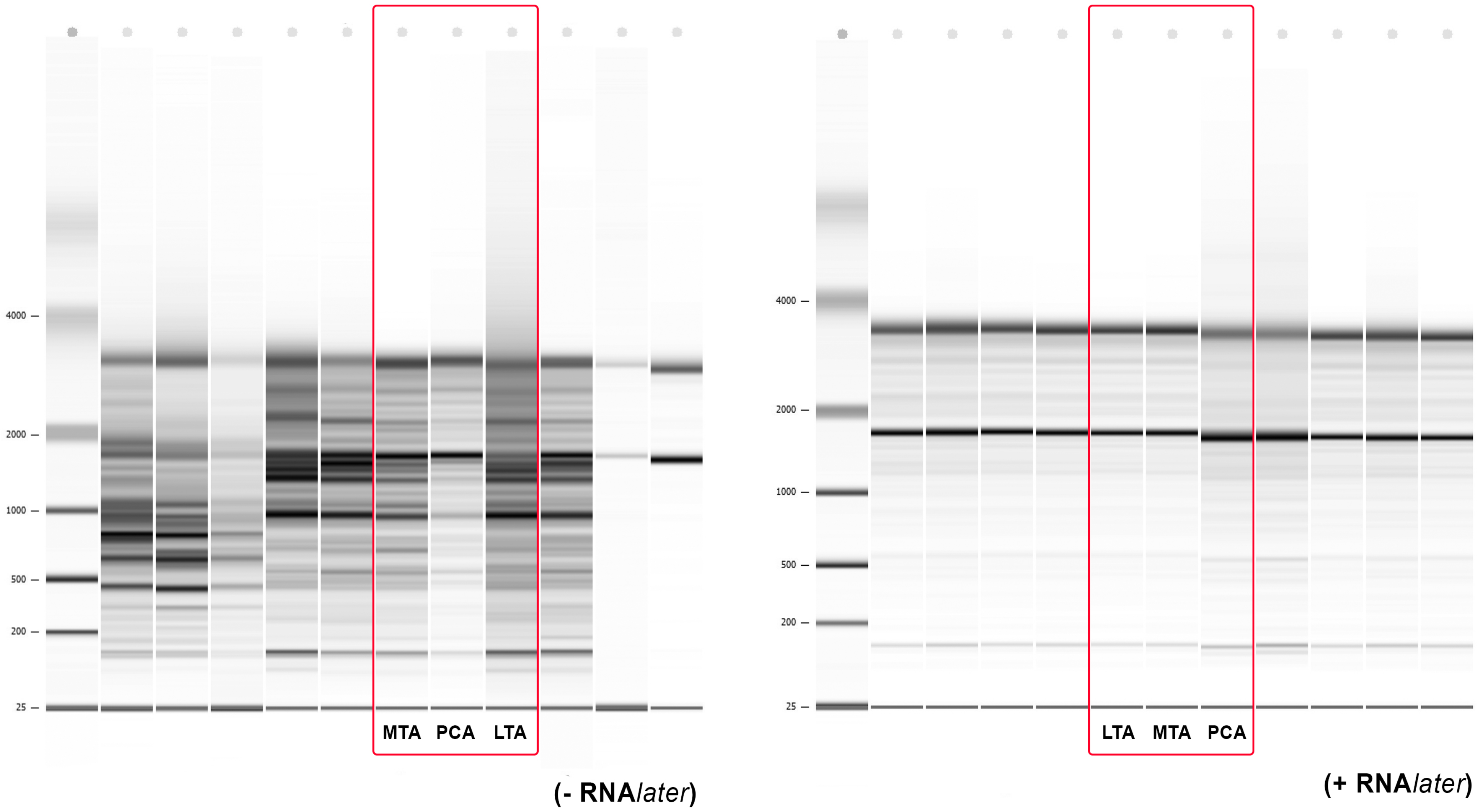

Supplementary Figure S1

Electrophoresis file run summary. Original gel electropherograms provided by Agilent 2100 Bioanalyzer Pico Chip were visualized using 5 µL total RNA. Gels before and after RNAlater application are shown as presented in Figure 2.

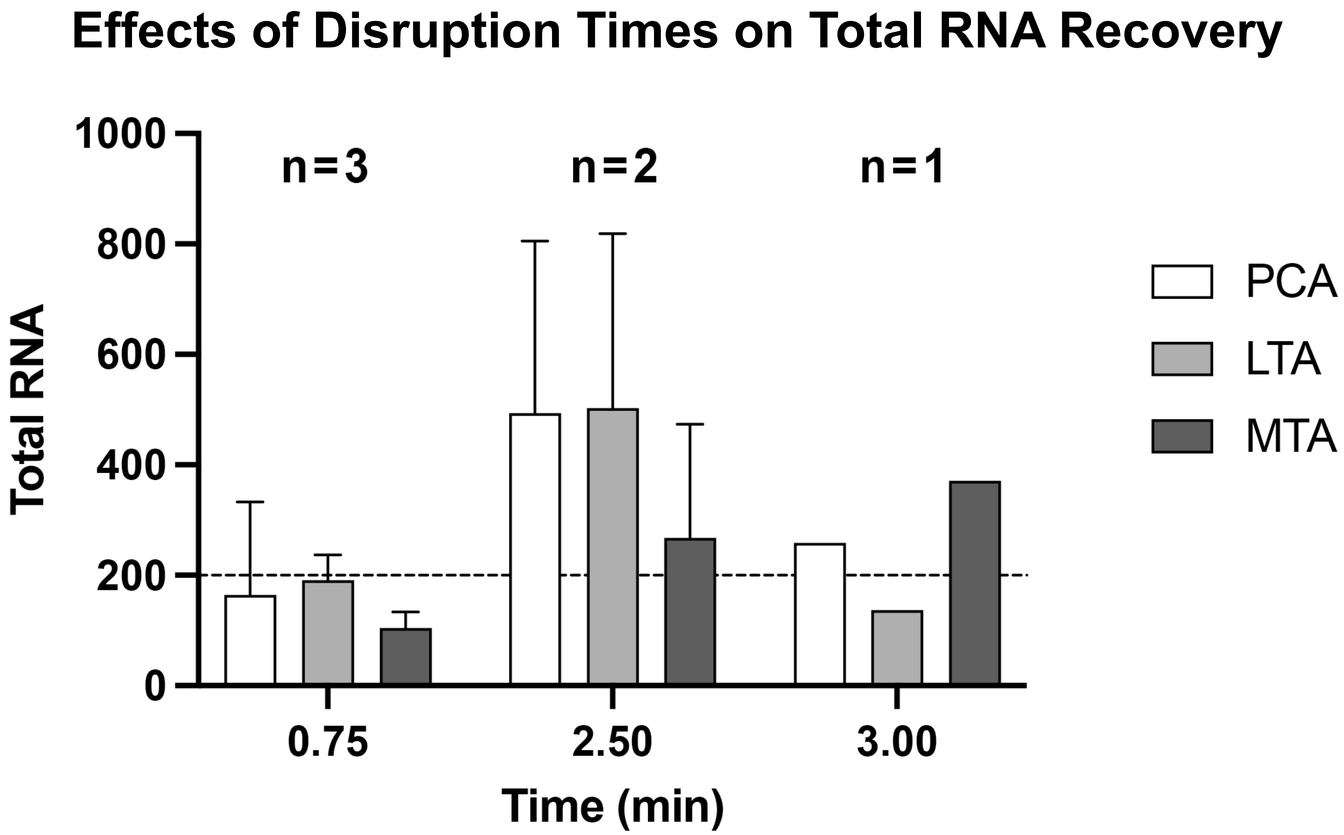

Supplementary Figure S2

Changes in RNA yield from varying rotor-stator durations (pt = 2), following the Aurum kit protocol. Total RNA (ng) was determined by the RNA concentration (ng/μL) measured via Nanodrop Spectrophotometer at 260 nm. The black dashed line denotes the target yield of RNA in this study (200 ng). Disruption times were assessed using 2 pooled muscle bellies to determine the most optimal rotor-stator disruption time associated with the recovered RNA concentration. The N is also noted to show how many trials were performed under each duration. Note, the 3 min disruption was only trialed once (no error bars shown) given that we quickly discerned that this period was excessive for most ILMs. Analysis of variance was performed using a two-way ANOVA with Posthoc tests. The data is expressed as mean±SD. * means $P < 0.05$; ** $P < 0.01$; *** $P < 0.001$; **** $P < 0.0001$.

Efficiency of the Proposed Extraction Method

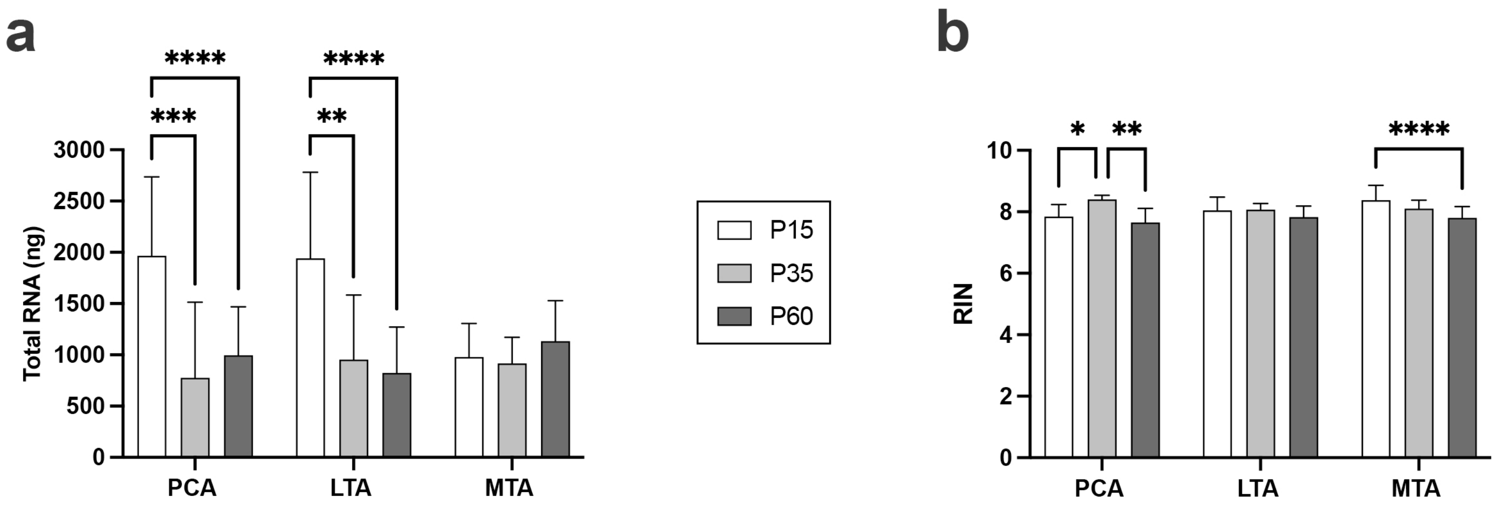

Supplementary Figure S3

Comparison of the experimental method at different rodent stages. The reproducibility of the protocol was confirmed by performing the proposed method at different stages. Note, the only changes to the optimized method were the number of pooled muscle bellies for sufficient RNA recovery, and corresponding rotor-stator disruption times based on the total weights of the PCA, LTA, and MTA muscles. (a) Differences in total RNA yield (provided by Nanodrop Spectrophotometer at 260 nm) between the P15 (pt =7), P35 (pt = 4), and P60 (pt =3) rats. (b) Significant changes were observed in the RIN, determined by Agilent 2100 Bioanalyzer Pico Chip. Analysis of variance was performed using a two-way ANOVA with Posthoc tests. The data is expressed as mean±SD. * means $P < 0.05$; ** $P < 0.01$; *** $P < 0.001$; **** $P < 0.0001$.
